# The Improvement of Semen Quality by Dietary Fiber Intake Is Positively Related With Gut Microbiota and SCFA in a Boar Model

**DOI:** 10.3389/fmicb.2022.863315

**Published:** 2022-05-11

**Authors:** Yan Lin, Ke Wang, Lianqiang Che, Zhengfeng Fang, Shengyu Xu, Bin Feng, Yong Zhuo, Jian Li, Caimei Wu, Junjie Zhang, Haoyu Xiong, Chenglong Yu, De Wu

**Affiliations:** ^1^Key Laboratory of Animal Disease-Resistance Nutrition and Feed Science, Institute of Animal Nutrition, Sichuan Agricultural University, Chengdu, China; ^2^Key Laboratory of Animal Disease-Resistance Nutrition, Ministry of Education, Chengdu, China; ^3^College of Life Science, Sichuan Agricultural University, Ya'an, China

**Keywords:** dietary fiber, growing boar, semen quality, gut microbiota, short-chain fatty acid

## Abstract

Although fiber-rich diets have been positively associated with sperm quality, there have not been any studies that have examined the effects of dietary fiber and its metabolites on sperm quality in young or pre-pubescent animals. In this study, we aimed to explore the effect of dietary fiber supplementation on semen quality and the underlying mechanisms in a boar model. Sixty purebred Yorkshire weaning boars were randomly divided into the four groups (T1–T4). Groups T1, T2, and T3 boars were fed diets with different levels of fiber until reaching 160 days of age and were then fed the same diet, while group T4 boars were fed a basal diet supplemented with butyrate and probiotics. Compared with T1 boars, sperm motility and effective sperm number were significantly higher among T3 boars. Meanwhile, at 240 days of age, the acetic acid and total short-chain fatty acid (SCFA) contents in the sera of T3 and T4 boars were significantly higher than those in T1 boars. The abundance of microbiota in T2 and T3 boars was significantly higher than that in T1 boars (*P* < 0.01). Moreover, dietary fiber supplementation increased “beneficial gut microbes” such as *UCG-005, Rumenococcus, Rikenellaceae_RC9_gut_group* and *Lactobacillus* and decreased the relative abundance of “harmful microbes” such as *Clostridium_sensu_stricto_1, Romboutsia* and *Turicibacter*. Collectively, the findings of this study indicate that dietary fiber supplementation improves gut microbiota and promotes SCFA production, thereby enhancing spermatogenesis and semen quality. Moreover, the effects of dietary fiber are superior to those of derived metabolites.

## Introduction

In recent years, dietary fiber has played an increasingly important role in the treatment of diabetes, obesity, colon cancer and other diseases by regulating the diversity and composition of intestinal flora. Dietary fiber intake has been reported to enhance the reproductive performance of pregnant women (Tomsett et al., 2020), rats (Lin et al., 2012), and sows (Zhou et al., 2017). However, a few studies conducted on males have shown that dietary fiber either has some beneficial effect on testosterone secretion and semen quality or no effect at all. Dorgan et al. (1996) found that plasma total testosterone and sex hormone-binding globulin levels were 13 and 15% higher, respectively, in the high-fat and low-fiber groups than in the low-fat and high-fiber groups in healthy men. Moreover, men with low-fat and high-fiber intake were reported to have reduced serum and urine androgen levels (Wang et al., 2005). Furthermore, studies in rabbits have shown that the addition of dietary fiber improves semen quality (Pascual et al., 2016). Thus, the findings of these studies have either directly or indirectly revealed that dietary fiber can have an important influence on reproductive hormone secretion and semen quality in male animals.

The intestine is the largest habitat for microorganisms and the composition and structure of the diet, especially fiber intake, has an important impact on the composition of intestinal flora (Mändar et al., 2015). Animals fed complex carbohydrate diets have a high abundance and diversity of gut flora. Inulin, composed of fructooligosaccharides, has been shown to increase the abundance of *Bifidobacteria* and reduce the number of total anaerobic bacteria and *Clostridium* in feces (Tian et al., 2017). Hermes et al. revealed that pectin in high-fiber diets is fermented by gut microbes, which increases short-chain fatty acid (SCFA) content and decreases intestinal pH. This inhibits the colonization and growth of certain pathogens and increases the number of beneficial bacteria (Hermes et al., 2009). Wu et al. (2018) fed weaned male piglets with different fiber sources and found that cellulose significantly increased the abundance of *Lactobacillus*, while xylan significantly increased the abundance of *Bifidobacterium*. This indicates that dietary fiber can regulate the structure of intestinal flora and that dietary fiber from different sources operates through various actions.

In addition to their associations with metabolic disorders, it is also well-established that components of the gut microbiota contribute to the regulation of reproductive hormone secretion (Hussain et al., 2021). For example, it has been demonstrated that dietary supplementation with probiotic *Lactobacillus reuteri* can increase and restore testosterone levels in aging mice; by using a testicular injury model, it was found that *Lactobacillus plantarum* TW1-1 has a regulatory effect on intestinal microbiota and can effectively ameliorate di(2-ethylhexyl) phthlate (DEHP)-induced testicular injury (Tian et al., 2019). Furthermore, exogenous supplementation with lactic acid, bacteria and bifidobacteria has been shown to enhance sperm motility and reduce the extent of sperm DNA fragmentation in asthenozoospermic human males (Valcarce et al., 2017), whereas in mice and broilers, probiotic supplementation enhances semen quality and sperm motility (Dardmeh et al., 2017; Inatomi and Otomaru, 2018). The aforementioned studies, thus, provide convincing evidence that gut flora has an important influence on the quality of semen in both the humans and animals. However, whether an intake of dietary fiber affects the composition of the gut microbiota and semen quality in young male animals still remains to be conclusively established.

Dietary fiber alters intestinal microorganisms, accompanied by changes in SCFAs. The major SCFAs in the colon and caecum of humans and pigs are acetic acid, propionic acid and butyric acid. Hermes et al. (2009) found that dietary fiber supplementation increased intestinal SCFA concentrations in 35-day-old piglets. Maternal fiber intake not only affected the production of SCFAs in sow feces, but also influenced the concentrations of SCFAs in the neonatal colon (Li et al., 2019a). In addition to providing energy, SCFAs are involved in regulating reproductive performance (Lin et al., 2014). Moreover, adding glyceride butyrate to the diet of cocks can improve sperm volume, sperm motility, sperm concentration and reduce abnormal sperm rates (Alhaj et al., 2018). Interestingly, exposing germ-free (GF) mice to *Clostridium tyrobutyricum* (CBUT), which secretes high levels of butyrate, increased their serum levels of gonadotropins [luteinising hormone (LH) and follicle-stimulating hormone (FSH)] (Al-Asmakh et al., 2014). Meanwhile, butyrate supplementation in suckling goat kids improved reproductive tract development and the total and daily gain by improving their antioxidant status (Mohamed et al., 2020). Therefore, we speculated that butyric acid, an important component of SCFAs, may play a role in regulating male reproduction.

We hypothesized that dietary fiber has beneficial effects on boar reproduction by inducing SCFAs production and regulating gut microbiota. Specifically, our objectives were to examine the effects and underlying mechanism of different levels of dietary fiber and their metabolites on semen quality in growing boars to provide a theoretical basis for dietary fiber applications in adolescent and immature male animals.

## Materials and Methods

### Experimental Design and Animals Used

All the animal procedures used in this study were approved by the Animal Care and Use Committee of Sichuan Agricultural University (SAU-ANI-2020-117). Sixty weaned purebred Yorkshire boars with similar weights were selected and randomly divided into the four groups: T1, T2, T3, and T4 (*n* = 15 each). The T1 group was fed a basic diet, whereas those in the groups T2 and T3 were fed diets containing different levels of dietary fiber; the T4 group was fed a basic diet supplemented with probiotics and tributyrin. The basic diets for each stage of growth for the boars were prepared according to the recommendations of the Nutrient Requirement of Swine from the National Research Council (NRC) (2012) and the nutrition levels are shown in Table 1. The supplementary fibers used were inulin and cellulose. In each stage, the amount of tributyrin (Perstorp, Shanghai Chemical Products Trading Corporation, Ltd, China) was added at a concentration of 0.6 g/kg of diet and *Lactobacillus* and *Bifidobacterium* cultures [3 × 10^8^ colony-forming unit (CFU)/g] were both added at 1 g/kg of diet. The growing boars received these diets until they reached an age of 160 days and then all the groups were fed the same diet. During the experimental period, the boars were provided with food and water *ad libitum* and their initial and final body weights were recorded.

**Table 1:** Calculated nutrient levels of diets.

**Nutrient** **composition**	**0–30 d**				**31–60 d**				**61–130 d**				**131–330 d**
	**T1**	**T2**	**T3**	**T4**	**T1**	**T2**	**T3**	**T4**	**T1**	**T2**	**T3**	**T4**	**All**
DE (Mcal/kg)	3.54	3.54	3.54	3.54	3.45	3.45	3.45	3.45	3.37	3.37	3.37	3.37	3.37
CP (%)	18.81	18.81	18.81	18.81	15.68	15.68	15.68	15.68	15.52	15.52	15.52	15.52	15.52
CF (%)	1.60	1.89	2.19	1.60	2.19	2.57	2.95	2.19	2.38	2.65	3.12	2.38	2.73
Ca (%)	0.81	0.81	0.81	0.81	0.68	0.68	0.68	0.68	0.84	0.84	0.84	0.84	0.82
AP (%)	0.48	0.48	0.48	0.48	0.34	0.34	0.34	0.34	0.32	0.32	0.32	0.32	0.33
ADF (%)	8.38	9.30	10.28	8.38	14.99	16.27	17.52	14.99	15.82	16.44	17.94	15.82	16.98
NDF (%)	2.80	2.98	3.19	2.80	5.83	6.13	6.42	5.83	6.01	6.16	6.50	6.01	6.36
SDF (%)	1.72	1.84	2.09	1.72	1.76	2.43	2.84	1.76	1.82	2.72	3.28	1.82	1.96
IDF (%)	8.25	9.63	10.88	8.25	10.92	12.75	14.84	10.92	11.57	14.17	17.11	11.57	12.85
TDF (%)	9.97	11.47	12.97	9.97	12.68	15.18	17.68	12.68	13.39	16.89	20.39	13.39	14.81

*T1, basal diet; T2, T3, basal diet supplemented with different fiber level; T4, basal diet supplemented with probiotics and butyrate. SDF, soluble fiber; IDF, insoluble fiber; TDF, total dietary fiber*.

To determine the beneficial effects of dietary fiber supplementation during the growing period, we assessed the quality of adult boar semen. At 210 days of age, the boars were trained to mount an artificial sows and semen was collected using the massage method from day 240 to day 330. After collection, semen volume, sperm concentration, and sperm motility were assessed according to previously reported methods (Ren et al., 2015). To minimize the effects of individual differences, eight ejaculations per boar were used to estimate sperm production. For microbiota analysis, we also collected fecal samples by massaging the rectum of boars to stimulate defaecation. The samples were immediately stored at −80°C until used for subsequent analyses.

### Growth Performance and Testicular Development

The daily feed intake of each boar was recorded, boars were weighed and the feed conversion ratio (F/G) was calculated based on body weight and feed intake. Each boar was scored for diarrhea at 08:00, 12:00, and 16:00 every day during the 0–30 days of the experiment. The diarrhea index was calculated as the sum of diarrhea scores of pigs per pen/(the number of pigs per pen × total days) (Le Floc'h et al., 2014). In addition, to assess the effect of dietary fiber supplementation on testis development, we measured the long and short testicular diameters on both the sides of each boar at 4-week intervals to determine testicular volume.

### Sexual Desire and Sperm Quality

The ejaculation response time and ejaculation duration of boars were measured (Louis et al., 1994). The sperm motility rate, sperm density and sperm motility characteristics of the filtered semen were determined using a computer-assisted sperm analysis (CASA) (Minitube, Tiefenbach, Germany) system. The total sperm count and effective sperm count per ejaculation were calculated using the formula described by Ren et al., 2015.

### Short-Chain Fatty Acid Measurement

The feces and serum of the boars were collected on the 120th and 240th day of the experiment, respectively. The SCFA content was determined by using a CP-3800 gas chromatography (Varian Incorporation, Walnut Creek, California, USA) according to the method described by Zhou et al., 2017.

### Bacterial Community Analysis

We adopted 16S rDNA sequencing technology to determine the microbial composition of feces collected on the 75th day of boar growth. Thirty-two fresh fecal samples collected from eight boars from the each treatment group were used to evaluate their respective microflora communities. Microbial community genomic DNA was extracted from the fecal samples using the E.Z.N.A.® Soil DNA Kit (Omega Bio-tek, Norcross, Georgia, USA) according to the manufacturer's instructions, with concentrations and purities of the isolated DNA being determined using a NanoDrop 2000 UV-Vis spectrophotometer (Thermo Fisher Scientific, Wilmington, USA). Using this DNA as a template, we amplified the V3–V4 hypervariable region of the bacterial 16S rRNA gene *via* an ABI GeneAmp® 9700 PCR Thermocycler (ABI, California, USA) using primer pair 338F (5′-ACTCCTACGGGAGGCAGCAG-3′) and 806R (5′-GGACTACHVGGGTWTCTAAT-3′). The purified amplicons were pooled in equimolar amounts and paired-end sequenced on an Illumina MiSeq PE300 platform/NovaSeq PE250 platform (Illumina, San Diego, California, USA) according to the standard protocols recommended by Majorbio Bio-Pharm Technology Corporation Ltd. (Shanghai, China).

The raw 16S rRNA gene sequencing reads, thus, obtained were demultiplexed, quality-filtered using fastp version 0.20.0 (Chen et al., 2018) and merged using FLASH version 1.2.7 (Magoč and Salzberg, 2011). Operational taxonomic units (OTUs) with a 97% similarity cutoff were clustered using UPARSE version 7.1 (Edgar, 2013) and chimeric sequences were identified and removed. The taxonomy of the representative sequence of each OTU was analyzed using RDP Classifier version 2.2 (Wang et al., 2007) against the 16S rRNA database (Silva v138) with a confidence threshold of 0.7. The Spearman's correlation analysis was used to assess the relationship between sperm quality and microorganisms.

### Statistical Analysis

Collected data were analyzed using the one-way ANOVA procedure of the Statistical Product and Service Solutions (SAS) statistical software (version 9.4; SAS Institute Incorporation, Cary, North Carolina, USA), followed by a generalized linear model (GLM) analysis. Values are expressed as mean ± SEM, with differences being considered statistically significant at *P* < 0.05 and a value of 0.05 ≤ *P* < 0.10 being indicative of a “tendency.”

## Results

### Growth Performance and Diarrhea

As shown in Table 2, the diarrhea indices of the T2, T3, and T4 groups were lower than those of the group T1 at all the stages, but the differences were not statistically significant (*P* > 0.05). Compared with the T1 boars, the diarrhea index of the T3 boars on days 0 to 14 showed a decreasing trend (*P* < 0.1). During the same period, the average daily feed intake of T4 boars was found to be significantly higher than that of the T3 boars (*P* < 0.05). However, the differences were not significant throughout the experimental period. Similarly, we detected no significant differences in the average daily gain or feed conversion ratio (*P* > 0.05).

**Table 2:** Effect of dietary fiber and metabolites on diarrhea and growth performance of boars.

**Items**	**T1**	**T2**	**T3**	**T4**	***P*-value**
**Diarrhea index**					
0–14 d	0.34 ± 0.07	0.25 ± 0.05	0.16 ± 0.04	0.20 ± 0.05	0.096
15–30 d	0.30 ± 0.10	0.30 ± 0.08	0.20 ± 0.07	0.25 ± 0.09	0.818
0–30 d	0.32 ± 0.07	0.27 ± 0.05	0.18 ± 0.05	0.22 ± 0.06	0.153
**Average daily gain, g**					
0–130 d	844.2 ± 21.2	839.6 ± 32	836.1 ± 30	860.3 ± 28.5	0.586
131–260 d	687.8 ± 25.8	649.9 ± 35.7	688.1 ± 24.3	685.0 ± 17.1	0.394
**Average daily feed intake, g**					
0–130 d	1.75 ± 0.04	1.75 ± 0.12	1.71 ± 0.09	1.78 ± 0.02	0.322
131–260 d	2.60 ± 0.04	2.58 ± 0.04	2.59 ± 0.06	2.60 ± 0.03	0.922
**Feed conversion ratio**					
0–130 d	2.07 ± 0.05	2.09 ± 0.10	2.04 ± 0.08	2.10 ± 0.07	0.322

*T1, basal diet; T2, T3, basal diet supplemented with different fiber level; T4, basal diet supple mented with probiotics and butyrate. Values are means and SEMs, n = 15 per group*.

### Testicular Development

**Figure 1:**
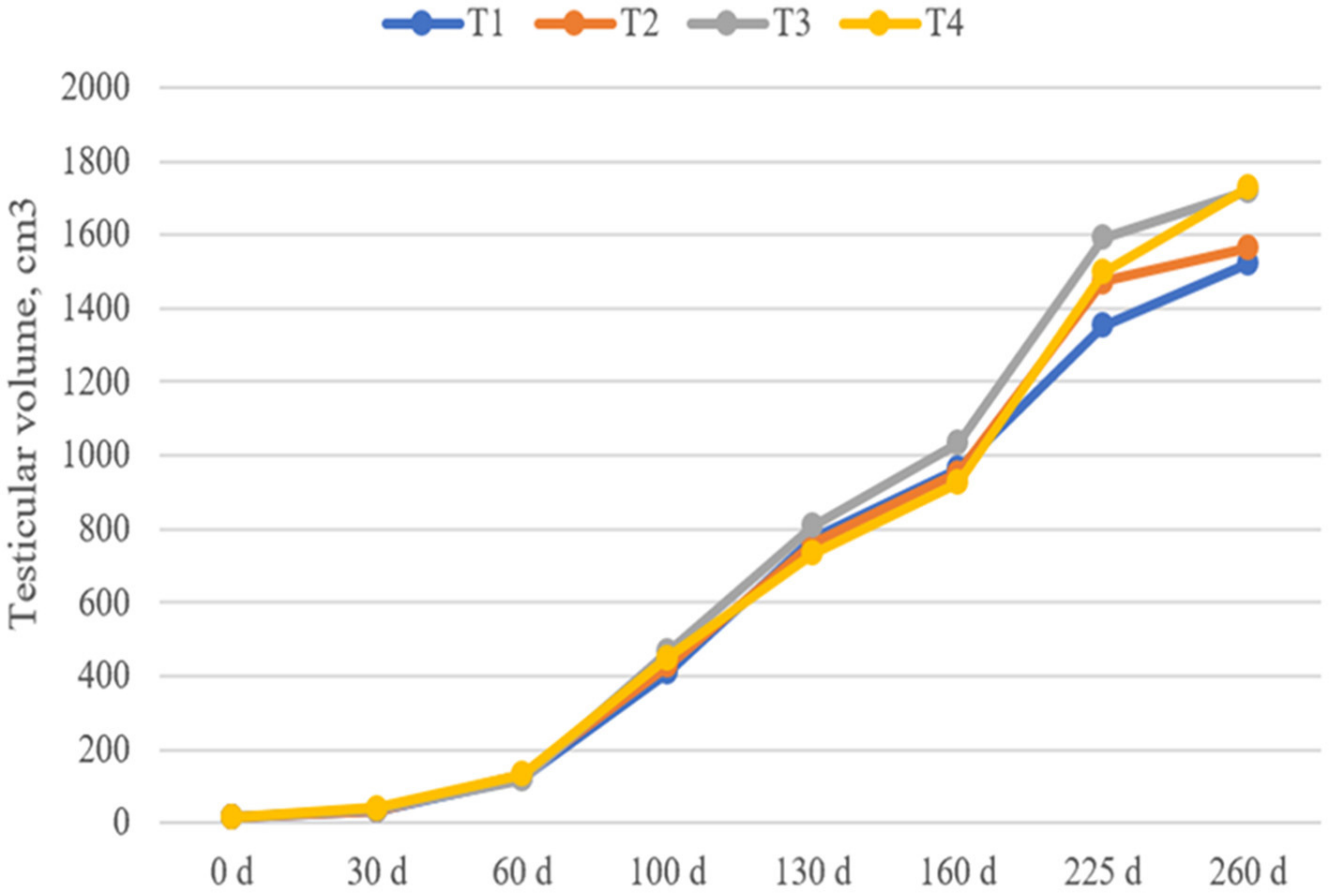
Effect of dietary fiber and metabolites on boar testicle volume. T1, basal diet; T2, T3, basal diet supplemented with different dietary fiber level; T4, basal diet supplemented with probiotics and butyrate. Values are means and SEMs, *n* = 15 per group.

As shown in Figure 1, although the testicular volumes of the T3 and T4 groups on days 225 and day 260 were 11–18 and 13–14% higher than those in the T1 group, respectively, there was no significant difference in the testis volume of boars among the groups at each stage (*P* > 0.05).

### Sexual Desire of Boars

As shown in Table 3, compared with the T1 group, the sexual desire of boars in the T4 group showed an increasing trend, although the difference was not statistically significant (*P* = 0.120). There was no significant difference in the ejaculation reaction time or duration among treatments (*P* > 0.05).

**Table 3:** Effect of dietary fiber and metabolites on libido of boars.

**Items**	**T1**	**T2**	**T3**	**T4**	***P-*value**
Libido score, s	3.42 ± 0.33	3.04 ± 0.45	3.24 ± 0.35	2.33 ± 0.33	0.120
Ejaculation reaction time, s	116.54 ± 37.56	115.23 ± 28.19	120.15 ± 40.11	78.69 ± 18.67	0.832
Duration of ejaculation, s	343.76 ± 68.82	375.66 ± 35.87	340.56 ± 13.23	483.91 ± 72.43	0.209

*T1, basal diet; T2, T3, basal diet supplemented with different fiber level; T4, basal diet supplemented with probiotics and butyrate. Values are means and SEMs, n = 6 per group*.

### Semen Quality of Boars

Notably, we detected significantly higher sperm motility in T3 boars during weeks 1–4 compared to T1 boars (*P* < 0.05); however, the proportion of immobile spermatozoa in T3 boars was lower than that in T1 boars (Table 4). Furthermore, T4 boars were found to have significantly higher sperm linear, oscillation, and forward ratios than group T1 boars (*P* < 0.01). There was no difference in the total sperm count per ejaculation during weeks 1–4 (Table 4). However, during weeks 5–8, there were marginally significant increase in sperm motility in T3 and T4 boars (*P* = 0.057; Table 5). In addition, during the entire assessed period (weeks 1–8), the motility of T3 boar sperm was significantly higher than that in the T1 and T2 boars (*P* < 0.05) and the effective sperm count per ejaculation showed an increasing trend (*P* = 0.068; Table 6). In addition, the sperm linear and oscillation ratios of T4 boars were significantly higher than those in T1 boars (*P* < 0.05).

**Table 4:** Effect of dietary fiber and metabolites on semen quality of boars (1–4 weeks).

**Items**	**T1**	**T2**	**T3**	**T4**	***P-*value**
Semen volume (mL)	84.65 ± 6.96	93.90 ± 6.83	97.35 ± 7.22	96.47 ± 10.06	0.951
Sperm density, ×10^8^ spz/mL	1.55 ± 0.11	1.53 ± 0.07	1.49 ± 0.12	1.47 ± 0.13	0.311
Sperm motility, %	87.38 ± 2.09	91.90 ± 1.27	94.46 ± 0.83	90.21 ± 2.53	0.028
Total sperm count/ejaculation, ×10^8^ spz	131.20 ± 10.11	143.67 ± 12.26	145.05 ± 9.37	141.80 ± 11.39	0.402
Effective sperm count/ejaculation, ×10^8^ spz	114.64 ± 6.16	132.03 ± 7.35	137.02 ± 8.24	127.92 ± 12.37	0.109
Immobile ratio, %	12.62 ± 2.09	8.10 ± 1.27	6.25 ± 1.06	9.79 ± 2.53	0.056
Linear ratio, %	33.16 ± 1.54^a^	33.24 ± 0.93^a^	30.69 ± 1.34^a^	39.00 ± 2.34^b^	0.005
Oscillation ratio, %	46.53 ± 1.23^a^	46.59 ± 0.75^a^	44.55 ± 1.07^a^	51.20 ± 1.88^b^	0.005
Forward ratio, %	70.57 ± 1.42^ab^	71.01 ± 0.87^ab^	68.33 ± 1.26^a^	75.31 ± 1.83^b^	0.009

*T1, basal diet; T2, T3, basal diet supplemented with different fiber level; T4, basal diet supplemented with probiotics and butyrate. Values are means and SEMs, n = 6 per group. Data with different letters are significantly different (p < 0.05)*.

**Table 5:** Effect of dietary fiber and metabolites on semen quality of boars (5–8 weeks).

**Items**	**T1**	**T2**	**T3**	**T4**	***P-*value**
Semen volume (mL)	113.70 ± 4.02	114.72 ± 5.68	125.60 ± 4.45	127.61 ± 6.73	0.174
Sperm density, ×10^8^ spz/mL	1.98 ± 0.09	1.90 ± 0.08	1.86 ± 0.09	1.80 ± 0.15	0.264
Sperm motility, %	91.10 ± 1.70	94.16 ± 1.61	95.28 ± 0.74	95.95 ± 1.37	0.057
Total sperm count/ejaculation, x10^8^ spz	225.39 ± 8.14	219.35 ± 9.75	233.61 ± 11.24	219.48 ± 7.48	0.162
Effective sperm count/ejaculation, ×10^8^ spz	211.57 ± 6.73^a^	199.07 ± 8.33^a^	222.58 ± 10.05^b^	220.59 ± 8.27^ab^	0.148
Immobile ratio, %	5.84 ± 1.70	8.90 ± 1.61	4.72 ± 0.74	4.05 ± 1.37	0.059
Linear ratio, %	33.96 ± 0.99	32.81 ± 1.56	35.44 ± 1.37	36.00 ± 1.64	0.406
Oscillation ratio, %	47.17 ± 0.79	46.25 ± 1.25	48.35 ± 1.09	48.80 ± 1.31	0.406
Forward ratio, %	71.80 ± 0.89	70.16 ± 1.45	72.74 ± 1.13	73.20 ± 1.61	0.357

*T1, basal diet; T2, T3, basal diet supplemented with different fiber level; T4, basal diet supplemented with probiotics and butyrate. Values are means and SEMs, n = 6 per group. Data with different letters are significantly different (p < 0.05)*.

**Table 6:** Effect of dietary fiber and metabolites on semen quality of boars (1–8 weeks).

**Items**	**T1**	**T2**	**T3**	**T4**	***P-*value**
Semen volume (mL)	99.17 ± 8.86	104.31 ± 6.36	116.47 ± 7.47	110.04 ± 7.58	0.414
Sperm density, ×10^8^ spz/mL	1.65 ± 0.09	1.67 ± 0.08	1.57 ± 0.09	1.64 ± 0.15	0.180
Sperm motility, %	90.22 ± 1.62^a^	91.53 ± 0.64^ab^	94.89 ± 1.37^b^	92.84 ± 1.71^ab^	0.026
Total sperm count/ejaculation, ×10^8^ spz	163.63 ± 7.05	174.20 ± 9.16	182.00 ± 11.13	180.46 ± 9.38	0.142
Effective sperm count/ejaculation, ×10^8^ spz	147.63 ± 6.78	159.44 ± 11.06	173.89 ± 9.35	167.54 ± 12.31	0.068
Immobile ratio, %	9.78 ± 0.52^b^	8.47 ± 0.70^b^	5.47 ± 0.64^a^	7.16 ± 0.59^ab^	0.049
Linear ratio, %	33.50 ± 0.97^a^	33.04 ± 0.87^a^	33.13 ± 1.02^a^	37.62 ± 1.48^b^	0.018
Oscillation ratio, %	46.80 ± 0.78^a^	46.43 ± 0.70^a^	46.50 ± 0.82^a^	50.10 ± 1.18^b^	0.018
Forward ratio, %	71.09 ± 0.90^ab^	70.62 ± 0.81^a^	70.59 ± 0.91^a^	74.35 ± 1.23^b^	0.037

*T1, basal diet; T2, T3, basal diet supplemented with different fiber level; T4, basal diet supplemented with probiotics and butyrate. Values are means and SEMs, n = 6 per group. Data with different letters are significantly different (p < 0.05)*.

### Short-Chain Fatty Acids in Feces and Serum of Boars

Analyses of the fecal contents of 120-day-old boars in each group revealed no significant difference in SCFA levels (*P* > 0.05; Table 7). However, we detected significantly higher concentrations of acetic acid and total SCFAs in the sera of 240-day-old boars from the groups T3 and T4 compared with those in T1 boars (*P* < 0.05). Furthermore, serum butyrate contents in T3 boars were significantly higher than those in the T1 and T2 boars (*P* < 0.05). However, no significant difference was observed among the groups with respect to propionic acid concentrations (*P* > 0.05).

**Table 7:** Effect of dietary fiber and metabolites on short-chain fatty acids (SCFAs) concentrations of boars.

**Items**	**T1**	**T2**	**T3**	**T4**	***P-*value**
**Feces in 120 Days**					
Acetate, mg/g	2.99 ± 0.14	3.37 ± 0.28	3.68 ± 0.39	4.02 ± 0.47	0.233
Propionate, mg/g	2.02 ± 0.12	2.46 ± 0.30	2.41 ± 0.27	2.36 ± 0.16	0.574
Isobutyric acid	0.26 ± 0.01	0.37 ± 0.06	0.35 ± 0.05	0.38 ± 0.04	0.336
Butyrate, mg/g	1.44 ± 0.11	1.82 ± 0.28	1.80 ± 0.29	1.86 ± 0.12	0.055
Isovaleric acid, mg/g	0.45 ± 0.02	0.71 ± 0.13	0.65 ± 0.11	0.73 ± 0.10	0.062
Valeric acid, mg/g	0.38 ± 0.02	0.68 ± 0.18	0.52 ± 0.1	0.51 ± 0.05	0.318
Total SCFAs, mg/g	7.54 ± 0.35	9.03 ± 1.14	9.41 ± 0.86	9.86 ± 0.88	0.075
**Serum in 240 days**					
Acetate, μmol/L	446.1 ± 49.3^a^	586.1 ± 22.0^ab^	687.3 ± 47.5^b^	660.8 ± 67.3^b^	0.014
Propionate, μmol/L	92.2 ± 6.1	87.79 ± 5.1	124.3 ± 20.4	101.1 ± 12.4	0.219
Butyrate, μmol/L	61.3 ± 13.7^a^	93.62 ± 10.7^a^	216.7 ± 39.7^b^	157.3 ± 27.9^ab^	0.003
Total SCFAs, μmol/L	599.7 ± 64.9^a^	767.6 ± 30.9^ab^	1028.0 ± 102.8^b^	919.1 ± 95.6^b^	0.008

*T1, basal diet; T2, T3, basal diet supplemented with different fiber level; T4, basal diet supplemented with probiotics and butyrate. Values are means and SEMs, n = 6 per group. Data with different letters are significantly different (p < 0.05)*.

### Overview of 16S rRNA Sequencing Data

To examine the effect of dietary fiber on the gut microbiota, stool samples were collected for 16S rRNA sequencing. A total of 1,400,127 sequences, with an average sequence length of 413 bp and 579,184,699 bases, were obtained from the 31 stool samples.

### Analysis of Microbial Diversity and Community Composition

**Figure 2:**
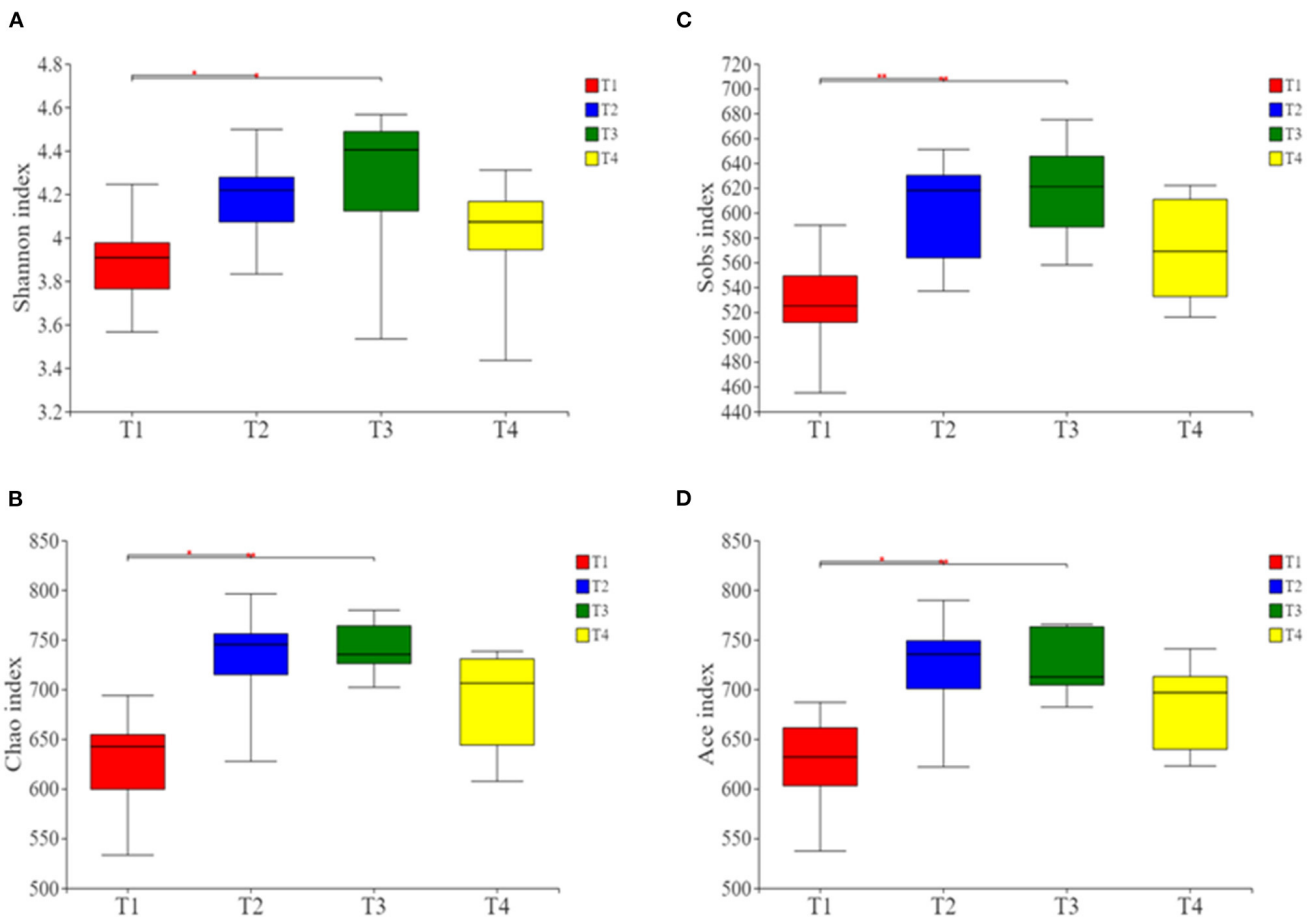
Alpha-diversity analysis of gut microbial community structure in each group. **(A)** Shannon index. **(B)** Chao index. **(C)** Sobs index. **(D)** Ace index. Red represents the T1 group, blue represents the T2 group, green represents the T3 group and yellow represents the T4 group. *p < 0.05, and **p < 0.01.

Alpha- and beta-diversity analyses revealed that compared with group T1 boars, the Shannon, Ace, Chao, and Sobs indices were significantly higher in T2 and T3 boars (*P* < 0.01; Figures 2A–D). This indicates that there were higher levels of microbial richness and diversity in the guts of boars fed a fiber-supplemented diet. Comparatively, boars fed with diets supplemented with butyrate and probiotic bacteria showed slight increase in gut microbiota diversity, indicating that the effects of metabolite supplementation are somewhat inferior to fiber supplementation.

**Figure 3:**
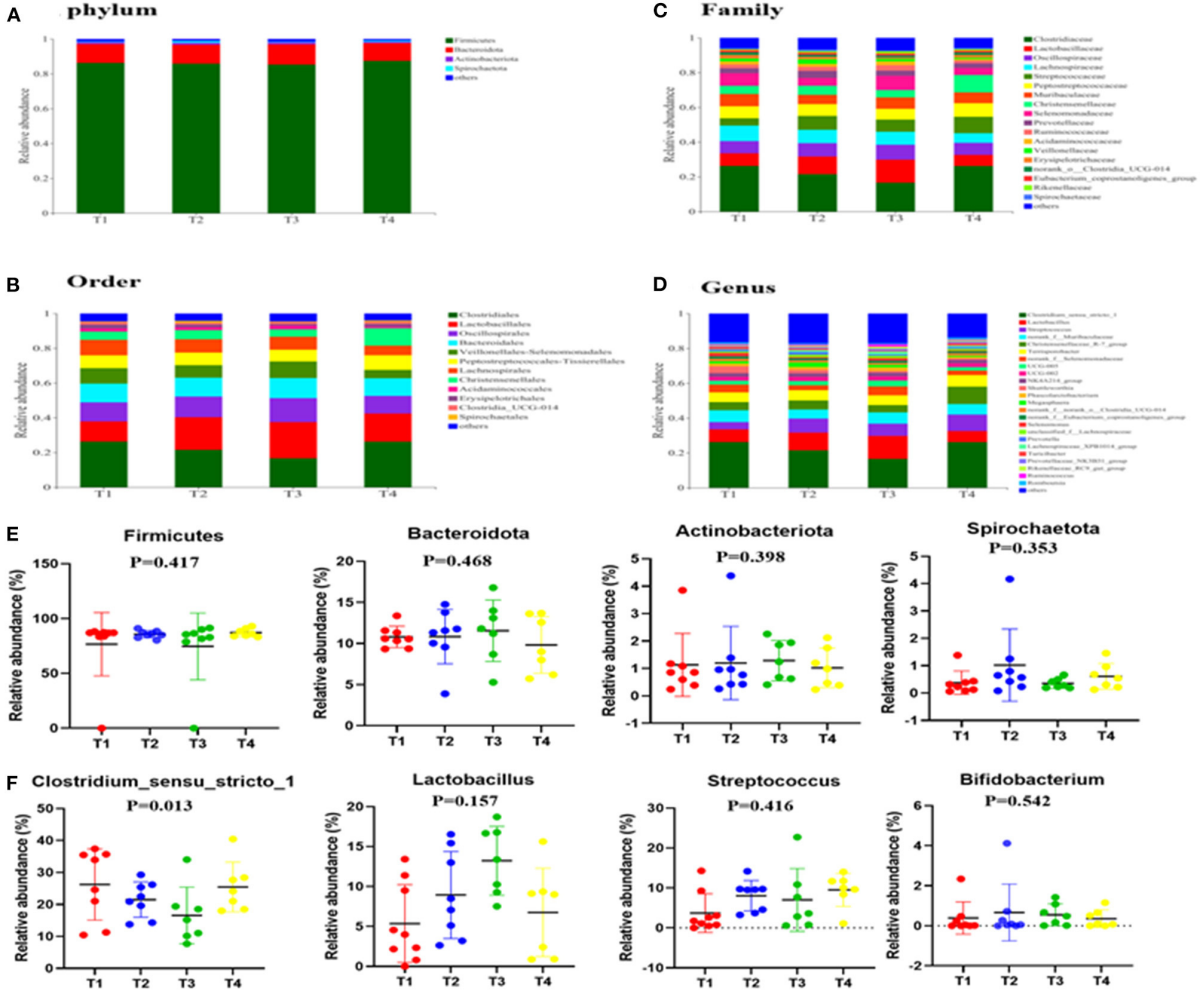
Effects of dietary fiber on fecal microbial composition. **(A,E)** The relative amounts of microbiota in feces at the phylum level. **(B)** The relative amounts of microbiota in feces at the order level. **(C)** The relative amounts of microbiota in feces at the family level. **(D,F)** The relative amounts of microbiota in feces at the genus level. Data were expressed as the mean ± SEM.

To gain insight into the effects of dietary supplementation on gut microbiota composition, we analyzed changes in bacterial community composition at the phylum, order, and genus levels (Figures 3A–F). Briefly, at the phylum level (Figures 3A,E), we established that *Firmicutes, Bacteroidetes* and *Actinobacteriota* were the three dominant phyla in all the four groups and that the abundance of *Cyanobacteria* in the guts of T3 boars (0.51%) was higher than that of T1 boars (0.190%). At the order level (Figure 3B), the abundances of *Clostridiales* and *Erysipelotrichales* in T3 boars were lower than those in T1 boars (*P* < 0.05), while the abundance of *Oscillospirales* was significantly higher than in T1 and T4 boars (*P* < 0.01). At the genus level (Figures 3D,F), the abundances of *Clostridium_*sensu_stricto_1, *Romboutsia* and *Turicibacter* were lower in T3 boars than in T1 boars (*P* < 0.05), while the abundances of UCG-005, *Ruminococcus, Rikenellaceae_*RC9_gut_group and *Lactobacillus* were higher in T3 boars (*P* < 0.05). Notably, the abundances of *Lactobacillus* increased in response to fiber and metabolite supplementation (*P* > 0.01), thereby providing evidence that dietary fiber has a potentially significant effect on gut microbiota composition.

### Differential Analysis of Gut Microbiota Changed by Dietary Fiber and Metabolites Supplementation

**Figure 4:**
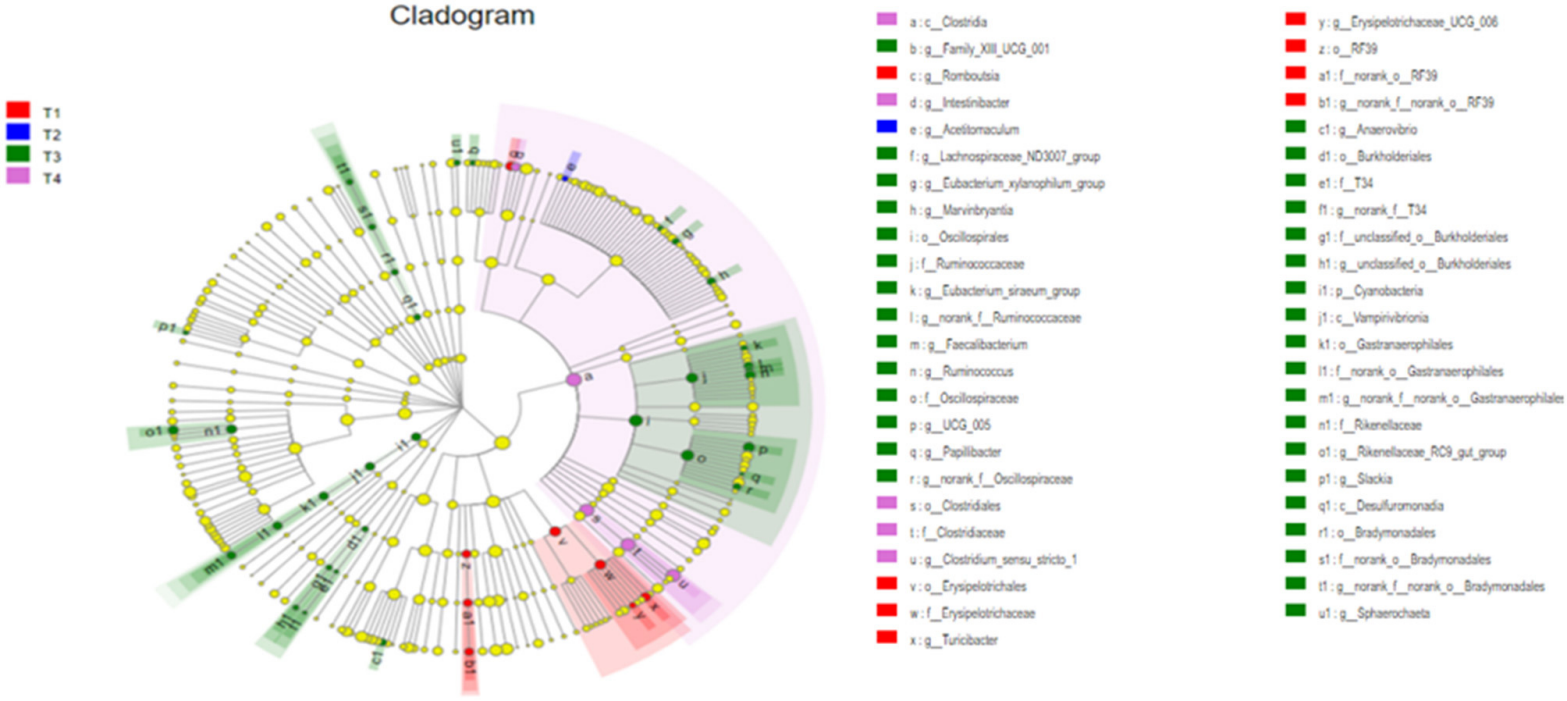
The differently abundant taxa identified using linear discriminant analysis effect size (LEfSe) analysis. The LEfSe cladogram shows the most differentially abundant taxa among the groups. Taxa enriched in the T1 group are red, taxa enriched in the T2 group are blue, taxa enriched in the T3 group are green and taxa enriched in the T4 group are purple. The size of each dot is proportional to its effect size.

To further screen for significant differences in the gut microbiota, differential analysis and linear discriminant analysis effect size (LEfSe) analysis were conducted. As shown in Figure 4, 213 taxa among the four groups were identified. The results indicate that the dominant bacteria in the T1 group were Erysipelotrichales, RF39, and *Romboutsia*; those in the T2 group were *Acetitomaculum*; those in the T3 group were Cyanobacteria, Desulfuromonadia, Oscillospirales, Burkholderiales, and Rikenellaceae; and in the T4 group was *Clostridia*.

### Relationships Between Sperm Quality and Intestinal Microbiota

**Figure 5:**
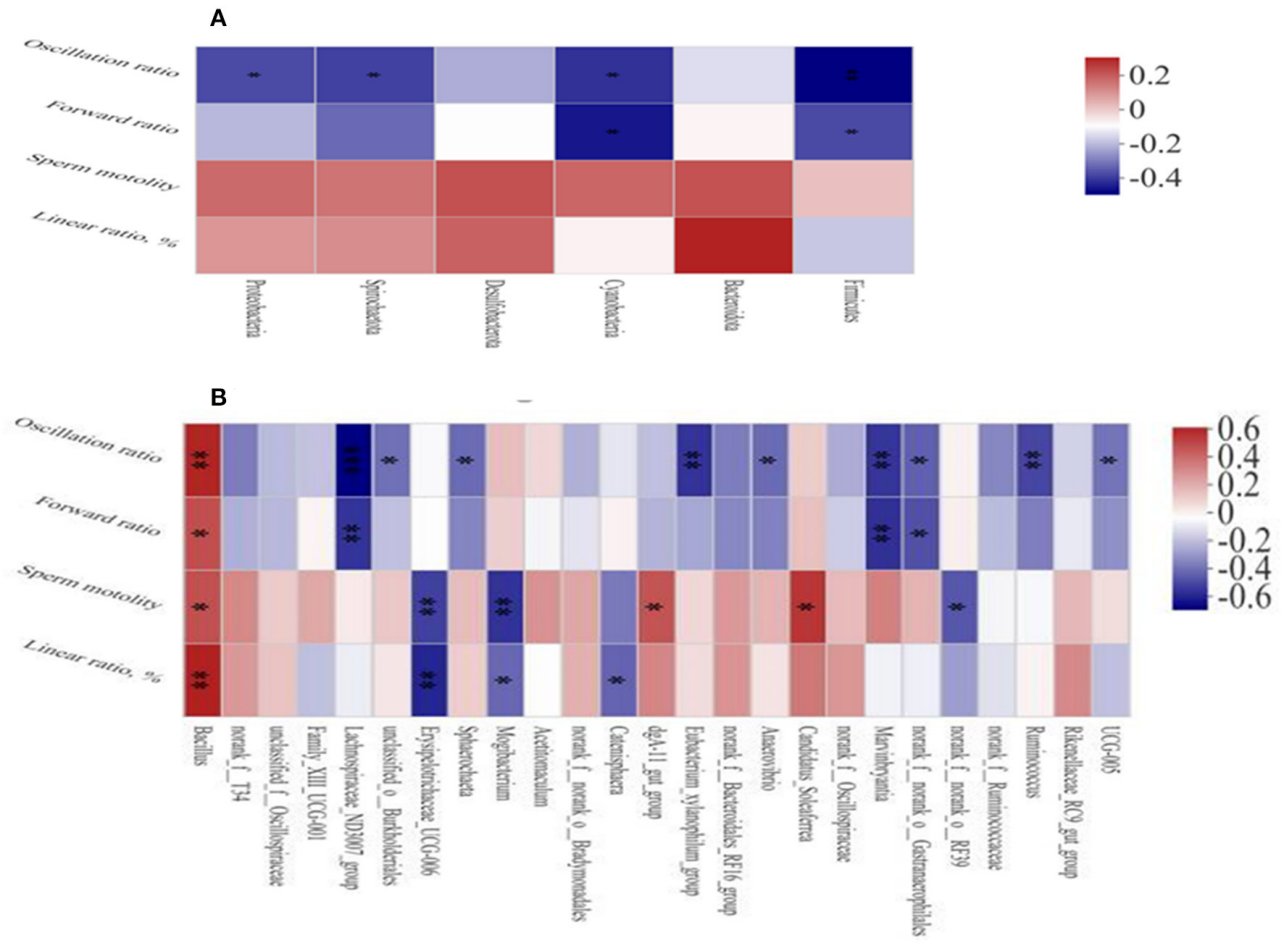
Heat map of correlation analysis between sperm quality and intestinal microbiota at the phylum **(A)** and genus **(B)** level. Spearman's diagram showed that the ordinate was environmental factor information and the abscess was species information. The value corresponding to the middle heat map was the Spearman's correlation coefficient r, which was between −1 and 1. *r* < 0 is negative correlation and *r* > 0 is positive correlation. *p < 0.05, **p < 0.01, and ***p < 0.001.

The Spearman's correlation analysis was used to study the relationship between environmental factors and microbial species richness and to study the relationship between environmental factors and species. At the phylum level, Firmicutes and Cyanobacteria were negatively correlated with the sperm forward ratio and Spirochaetota (Figure 5A), Proteobacteria, Firmicutes, and Cyanobacteria were negatively correlated with the sperm oscillation ratio. At the genus level (Figure 5B), *Bacillus Candidatus_Soleaferrea* and *dgA-11gut_group* were positively correlated with sperm motility and *Bacillus* was positively correlated with sperm motility, oscillation ratio and linear ratio. Furthermore, *Mogibacterium*, Erysipelotrichaceae_UCG-006 and norank_f_norank_o_RF39 were negatively correlated with sperm motility. The *Lachnospiraceae_ND3007_group, Ruminococcus, Marvinbryantia*, and *Eubacterium_xylanophilum_group* were negatively correlated with the sperm oscillation ratio.

## Discussion

In this study, dietary fiber supplementation had no significant effect on the average daily gain, average daily feed intake and feed conversion ratio of Yorkshire boars at each growth stage. Although dietary fiber and metabolites tended to reduce the rate of early diarrhea, they had no significant effect on growth performance throughout the study period. This finding is supported by Batson et al. (2021), who demonstrated that piglets fed different fiber source had no change in growth performance, but pigs fed added cellulose had increased fecal DM. In addition, these findings suggest that DF treatment could reduce the diarrhea rate in piglets, which is consistent with the findings of Shang et al. (2021). The results of this study showed that the testes exhibited temporal and spatial changes with the development of growing boars.

Few studies have examined the effects of fiber on testicular development. In this study, we also established that dietary fiber supplementation was associated with the temporal and spatial changes in the testicular development of growing boars. On day 260, the testicular volumes of group T3 and T4 boars increased by 13–14% compared to the control group boars, but were not significant. Our previous studies have shown that maternal fiber intake can increase the number of spermatogonial stem cells in postnatal boars (Lin et al., 2019), indicating that appropriate dietary fiber levels may promote testicular development; however, this needs to be verified by further studies.

The sperm motility and effective sperm viability of boars in the T3 group increased significantly after supplementation with dietary fiber, while the sperm linear ratio and forward ratio increased significantly after the addition of tributyrin and probiotics. This suggests that dietary fiber and metabolite supplementation before sexual maturation have an important effect on spermatogenesis and sperm quality in adult boars. Very few studies have demonstrated the direct effect of fiber on sperm quality. Braga et al. (2012) observed that a diet comprising cereals and vegetables was positively associated with man sperm quality. Male rabbits fed a diet enriched with soluble fiber had less sperm abnormalities and higher percentages of normal and motile spermatozoa (Pascual et al., 2016). Moreover, study on women showed that partially hydrolysed guar gum supplementation helped to improve gut dysbiosis and pregnancy success in women with infertility (Komiya et al., 2020). Consistently, dietary fiber supplementation has been shown to improve the reproductive performance of sows (Li et al., 2019b). Collectively, these foregoing findings provide convincing evidence to indicate that dietary fiber supplementation has potentially beneficial effects on the reproductive performance of both the male and female boars.

Short-chain fatty acids are the main metabolites generated from the microbial fermentation of dietary fiber in the gut. In this study, total SCFAs, acetate and butyrate significantly increased after dietary fiber or metabolite supplementation. Butyrate is one of the main metabolites in the intestinal microbial fermentation of dietary fiber. In addition, previous studies have established that supplementing the diets of adult roosters with sodium butyrate can improve semen volume and sperm motility by enhancing antioxidant capacity and testosterone hormone secretion (Alhaj et al., 2018). Butyric acid, which acts as a potent inhibitor of histone deacetylase (HDAC), has been reported to be involved in modulating sperm movement (Parab et al., 2015) and is a key regulator of the transcriptional program during the meiotic-to-post-meiotic transition in spermatogenesis (Yin et al., 2021). Furthermore, butyrate plays an important role in the redox balance (Lin et al., 2014), which is very important for spermatogenesis (Sênos and Jones, 2021). In addition, SCFAs have been found to exert notable immune and anti-inflammatory effects. In mice, butyrate was shown to promote the secretion of regulatory T cells (Tregs), interleukin-18 (IL-18) and the production of interleukin-10 (IL-10) in intestinal epithelial cells (Singh et al., 2014). SCFAs have also been demonstrated to activate NLRP3 inflammasome and decrease the inflammatory response in the gut (Macia et al., 2015). In this study, we observed that although butyrate supplementation promoted increase in the concentrations of total SCFAs, acetate and butyrate in group T4 boars, the levels were lower than those recorded in T3 boars, which indicate that the beneficial effects of butyric acid supplementation are less pronounced than diets supplemented with fiber. An increasing number of studies have shown that probiotics play a very important role in regulating body health and reproduction. Rats supplemented with a prebiotic, mannan-oligosaccharides (MOS), showed higher concentrations of testosterone and increased spermatozoa relative scores of the testicles (Rodrigues et al., 2021). Furthermore, supplementation of a probiotic with a prebiotic improved the quality/quantity of spermatozoa in infertile patients (Maretti and Cavallini, 2017). From the abovementioned results, it can be concluded that dietary fiber supplementation has beneficial effects on spermatogenesis and the actions of butyrate, *Lactobacillus*, and Bifidobacterium supply are inferior to those of dietary fiber.

It has been reported that the gut microbiota thrives on fiber-rich diets and, thus, the addition of fiber (or metabolites) to diets has a great effect on microbiota composition in humans and animals (Li et al., 2019b). In this study, the relative abundance of *Clostridium_sensu_stricto_1* decreased, whereas that of *Lactobacillus* tended to increase with the addition of dietary fiber. A previous study showed that high-purity insoluble dietary fiber supplementation affected the proliferation of key bacteria, such as *Lactobacillus* and Peptostreptococcaceae and then affected the synthesis of SCFAs in mice (Lyu et al., 2022). Dietary fiber supplementation increased the abundance of *UCG-005* in sows (Yu et al., 2020) and *Lactobacillus* in rats (Hua et al., 2021) and decreased the abundance of *Turicibacter i*n sows (Yu et al., 2021). Consequently, the addition of fiber to diets would appear to contribute to maintaining and enhancing the balance of intestinal bacterial numbers and diversity.

Moreover, the effects of *Lactobacillus* and *Bifidobacterium* supplementation on flora composition and abundance were inferior to those of dietary fiber supplementation. Study has shown that supplementation with a mixed prebiotic blend elicited the most abundant changes in microbiota, fermentative end products and immunoglobulin A (IgA) in dogs (Panasevich et al., 2021). However, there is limited study on the effect of dietary fiber supplementation on the microbiota composition in male animals. A high-fat diet induces intestinal flora disorders, resulting in testicular dysfunction and decreased spermatogenesis (Ding et al., 2020). In addition to this, *Lactobacillus* species significantly reversed testicular damage induced by gamma irradiation in rats (Changizi et al., 2021). *Lactobacillus casei* and *Lactobacillus coagulans* supplementation have protective effects against CCl_4_-induced testicular toxicity, thereby increasing the rate of spermatogenesis in rats (Keshtmand et al., 2021). Meanwhile, probiotic supplementation has been reported to exert beneficial effects on semen quality (Tomaiuolo et al., 2020). A 6-week long period of supplementation with *Lactobacillus* and *Bifidobacterium* in asthenozoospermic males increased sperm motility and reduced the rate of sperm DNA fragmentation (Valcarce et al., 2017). Moreover, a 6-month long treatment with daily administration of *Lactobacillus paracasei*, arabinogalactan, fructo-oligosaccharides and L-glutamine had a positive effect on sperm count and motility (Maretti and Cavallini, 2017). Furthermore, fecal microbiota transplantation (FMT) from alginate oligosaccharide (AOS)-dosed animals improved mouse sperm quality and spermatogenesis after busulfan treatment (Zhang et al., 2021). As mentioned above, dietary fiber and metabolites affect microbiota composition, diversity and sperm quality; therefore, it seems that gut microbiota might also be involved in the beneficial effects of dietary fiber on spermatogenesis.

Due to the lack of study on the results so far, the effect of microbiota on spermatogenesis is still unclear. In this study, we found that there was significantly correlation between the microbiota and sperm motility and oscillation ratio. Study has shown that glyphosate-induced gut microbiota dysbiosis changes the relative abundance of the phyla Bacteroidetes and Firmicutes and reduces sperm motility, while increasing the sperm malformation ratio in rats (Liu et al., 2022). With the increase in the genera *Bacteroides* and *Prevotella*, semen production decreased after the intake of a high-fat diet (Ding et al., 2020). *Lactobacillus* seemed to play a beneficial role in semen quality as *Lactobacillus* supplementation in rats increased the rate of spermatogenesis, which was accompanied by significant increase in the testicular spermiogenesis index (Keshtmand et al., 2021). Collectively, these findings reveal strong correlations between gut microbiota and spermatogenesis. To the best of our knowledge, this study is the first study to report that supplementing pig diets with fiber prior to sexual maturity could represent an effective strategy for enhancing spermatogenesis and sperm motility by improving the balance of the gut microbiota and promoting SCFA production. Further study is necessary to reveal the respective roles of the microbiota and SCFAs in male reproduction.

## Conclusion

In summary, we demonstrated that fiber supplementation in boar diets prior to their sexual maturity improves semen quality by favorably modifying gut microbiota composition and promoting the production of SCFAs. Moreover, the effects of dietary fiber were superior to those obtained by diets supplemented with dietary fiber metabolites. Our findings indicate that dietary fiber supplementation prior to puberty should be considered a potential nutrition-centered approach for improving animal reproduction and that of humans.

## Data Availability Statement

The original contributions presented in the study are publicly available. This data can be found at: https://www.ncbi.nlm.nih.gov/bioproject/, PRJNA814434.

## Ethics Statement

The animal study was reviewed and approved by Animal Care and Use Committee of Sichuan Agricultural University (SAU-ANI-2020- 117).

## Author Contributions

DW and YL conceived and designed the study. YL, HX, KW, LC, ZF, SX, and CY performed the experiments and analyzed the data. BF, JL, YZ, and CW contributed to the laboratory analysis. YL and JZ wrote and edited the original draft. All authors have read and agreed to the published version of the manuscript.

## Funding

This study was funded by the National Key R&D Program of China (2018YFD0501002), the National Natural Science Foundation of China (Grant No. 035Z2060), and the Sichuan Pig Innovation Team (sccxtd-2022-08).

## Conflict of Interest

The authors declare that the research was conducted in the absence of any commercial or financial relationships that could be construed as a potential conflict of interest.

## Publisher's Note

All claims expressed in this article are solely those of the authors and do not necessarily represent those of their affiliated organizations, or those of the publisher, the editors and the reviewers. Any product that may be evaluated in this article, or claim that may be made by its manufacturer, is not guaranteed or endorsed by the publisher.
